# A Novel High-Throughput Vaccinia Virus Neutralization Assay and Preexisting Immunity in Populations from Different Geographic Regions in China

**DOI:** 10.1371/journal.pone.0033392

**Published:** 2012-03-16

**Authors:** Qiang Liu, Weijin Huang, Jianhui Nie, Rong Zhu, Dongying Gao, Aijing Song, Shufang Meng, Xuemei Xu, Youchun Wang

**Affiliations:** 1 Department of Cell Biology, National Institutes for Food and Drug Control, Key Laboratory of the Ministry of Health for Research on Quality and Standardization of Biotech Products, Beijing, China; 2 Department of Biophysics and Structural Biology, Institute of Basic Medical Sciences Chinese Academy of Medical Sciences, School of Basic Medicine Peking Union Medical College, Beijing, China; 3 Beijing Blood Center, Beijing, China; Universidade Federal de Minas Gerais, Brazil

## Abstract

**Background:**

Pre-existing immunity to Vaccinia Tian Tan virus (VTT) resulting from a large vaccination campaign against smallpox prior to the early 1980s in China, has been a major issue for application of VTT-vector based vaccines. It is essential to establish a sensitive and high-throughput neutralization assay to understand the epidemiology of Vaccinia-specific immunity in current populations in China.

**Methodology/Principal Findings:**

A new anti-Vaccinia virus (VACV) neutralization assay that used the attenuated replication-competent VTT carrying the firefly luciferase gene of *Photinus pyralis* (rTV-Fluc) was established and standardized for critical parameters that included the choice of cell line, viral infection dose, and the infection time. The current study evaluated the maintenance of virus-specific immunity after smallpox vaccination by conducting a non-randomized, cross-sectional analysis of antiviral antibody-mediated immune responses in volunteers examined 30–55 years after vaccination. The rTV-Fluc neutralization assay was able to detect neutralizing antibodies (NAbs) against Vaccinia virus without the ability to differentiate strains of Vaccinia virus. We showed that the neutralizing titers measured by our assay were similar to those obtained by the traditional plaque reduction neutralization test (PRNT). Using this assay, we found a low prevalence of NAb to VTT (7.6%) in individuals born before 1980 from Beijing and Anhui provinces in China, and when present, anti-VTT NAb titers were low. No NAbs were detected in all 222 samples from individuals born after 1980. There was no significant difference observed for titer or prevalence by gender, age range and geographic origin.

**Conclusion:**

A simplified, sensitive, standardized, reproducible, and high-throughput assay was developed for the quantitation of NAbs against different Vaccinia strains. The current study provides useful insights for the future development of VTT-based vaccination in Beijing and Anhui provinces of China.

## Introduction

Vaccinia Tian Tan virus (VTT) was historically used for the vaccination of millions of Chinese people during the worldwide smallpox prevention campaign, and such programs led to the eradication of Variola in China prior to 1980 [1]–[4]. In recent years, VTT has been used as a virus vector for the development of potential vaccines for Human immunodeficiency virus (HIV), Hepatitis B virus (HBV), Human papillomavirus (HPV), Influenza virus subtype H5N1, Aichi virus (AIV), Severe acute respiratory syndrome coronavirus (SARS-CoV), and Rabies virus that can confer protection to immunized animals [5]–[11]. Although these approaches have been successful in animal models, significant problems remain for the use of VTT vector in humans. Current views on Vaccinia virus (VACV) suggest that it’s immunity is high or existent in the current population born before the early 1980s [12], which may influence both the titer and duration of the antibody response induced by a second distinct Vaccinia recombinant vaccine [13]. Preexisting immunity to viral vectors has been a major issue for the application of VTT vector-based vaccines in humans [14]. Therefore, a high-throughput neutralization assay is urgently needed for assessing the level of immunity to VACV in current populations. Such a neutralization assay would also be useful to monitor the efficiency of vaccination in experimental and clinical settings and allows standardization worldwide.

The conventional method used for determining anti-Vaccinia neutralizing antibody titer is the plaque reduction neutralization test (PRNT). The PRNT is considered the “gold standard” of assays because it is specific, direct, and reproducible [15], [16]. However, the PRNT is time-consuming and labor-intensive, which is not applicable for the large-scale screening that is needed for a population survey. In recent years, there have been several assays developed that are high-throughput, semi-automated, and do not rely on plaque formation and manual counts. Some of these assays detect aggregate cell infection as indicated by enzyme immunoassay [17] or the expression of recombinant reporter genes, such as β-galactosidase (β-gal) and green fluorescent protein (GFP) [18], [19]. Perceived difficulties of these assays may include the use of cell suspension cultures for GFP assays, which may be laborious to maintain, and a lower dynamic range observed with enzymatic (BGZ) assays.

Here, we describe the development of a novel neutralization assay in a 96-well format that uses the replication-competent VTT possessing a firefly luciferase protein reporter gene (rTV-Fluc). Upon infection, neutralizing antibody (NAb) activity is evaluated as a function of the reduction of the Fluc activity in the presence of specific anti-Vaccinia antibodies in the serum. The use of Fluc in the neutralization assay has several advantages over other reporters, such as, chloramphenicol acetyltransferase (CAT), β-gal, and GFP, including high sensitivity, broad dynamic range, and simplicity [20]. The sensitivity of chemiluminescence detection has been reported to be 10-fold greater than a fluorescence-based assay, and 80- to 100-fold greater than colorimetric methods [21].

Since people who were vaccinated decades ago with Vaccinia still maintain some immunity against VTT, future vaccination using VTT vector-based vaccines might be affected. Thus, it is important to clarify whether individuals vaccinated decades ago maintain any immunity to VTT, and if so, what proportion of the population possesses this immunity and the effectiveness of this immunity. The current study used the novel rTV-Fluc neutralization assay to study the prevalence of VTT-specific antibodies among a representative current Chinese population.

## Results

### Construction of rTV-Fluc

After approximately 6–10 rounds of purification, insertion of the Fluc into the VTT genome was confirmed by polymerase chain reaction (PCR), and expression was detected using the chemiluminescence assay. The amplified Fluc product was identified that exactly matched the published sequence (GenBank accession: EU921841). The 10 mL rTV-Fluc virus stocks (lot number: V201135) with a titer of 10^7^ plaque-forming units (PFU)/mL were aliquoted and stored at liquid nitrogen at China Center For Type Culture Collection, Wuhan, China.

### Optimization of conditions for the neutralization assay

**Figure 1:**
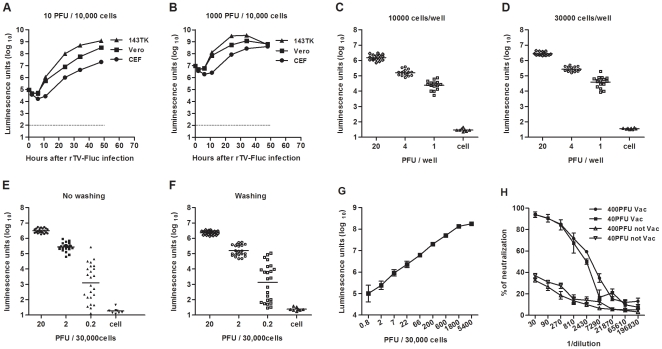
The optimization of the rTV-Fluc neutralization assay. (A-B) Several cell lines were compared for use in the luciferase assay. The x-axis indicates hours after rTV-Fluc infection, added to 10 PFU/30,000 cells (A) and 10^3^ PFU/30,000 cells (B) in a 96-well plate. The dotted line represents the background level of three cell lines. (C–D) The determination of Vero cell density. The x-axis indicates rTV-Fluc input, added to 10^4^ cells/well (C) and 3×10^4^ cells/well (D) in a 96-well plate. The dot shows the luciferase transgene expression after 24 h incubation. (E–F) The influence of washing with PBS before the addition of luciferin substrate. The x-axis indicates rTV-Fluc input, and the dot represents the luciferase transgene expression after 24 h incubation with no washing (E) and washing (F). (G) Dose-response between rTV-Fluc input and luciferase transgene expression. The x-axis indicates virus concentration, ranging from 0.8–5400 PFU/well, added to 3×10^4^ cells/well in a 96-well plate. Each data point represents the average of eight experiments. (H) Two different concentrations of rTV-Fluc (•,▴, 400 PFU/well; ▪,▾, 40 PFU/well) were used with various concentrations of rabbit serum for neutralizing activity. The same experiment was carried out with rabbit sera collected before (▴,▾) and two weeks after (•,▪) vaccination with VTT. Each data point represents the average of three experiments.

Several cell lines were compared for use in the Fluc assay with different doses of rTV-Fluc virus (Figure 1A and B). Luciferase expression was detected by incubation with luciferin substrate. The two cell lines, Vero and 143TK, produced very low background similar to the control CEF cells (not shown). It was found that 80% of 143TK cells resulted in cytopathic effect after infection for 36 h in the high dose group (10^3^ PFU/10^4^ cells in a 96-well plate). Therefore, the 143TK cell line was not suitable for this assay due to the high susceptibility to rTV-Fluc infection. Thus, the Vero cell line was chosen for further experiments because susceptibility was less than for 143TK cells, and it was suitable for low-titer NAb detection. Since Vero cells had a slow growth rate, 10^4^ cells/well in a 96-well plate were needed to produce a monolayer that allowed detection of viral proliferation following a 2-day incubation. Shorter incubation periods were examined and a 24 h incubation period was sufficient to distinguish the luciferase expression of infected cells from the background level of normal cells, 87.1-fold higher than background. Moreover, comparison of the addition of 10^4^ cells/well and 3×10^4^ cells/well in a 96-well plate favored the higher cell density due to better reproducibility (CV, 3.07% vs 1.75%, at 20 PFU/well; Figure 1C and D). It was found that washing once with phosphate-buffered saline (PBS) before the addition of the luciferin substrate did not significantly decrease the luciferase antigen expression compared with no washing (Figure 1E and F).

Vero cells were infected with different doses of rTV-Fluc to determine the optimal concentration to be used in the neutralization assay. Figure 1G depicts the direct correlation between rTV-Fluc concentration and luciferase transgene expression. There was a dynamic range of viral infecting dose (22–1800 PFU/30,000 cells), where the increase in expression was linearly correlated with the increase in viral infection dose. Next, the neutralizing activity of a Vaccinia-vaccinated rabbit serum sample was determined comparing different rTV-Fluc concentrations to evaluate the optimal multiplicity of infection (MOI) of virus to be used in this assay (Figure 1H). Poor virus neutralization was observed at PFU/well cell ratios of 40 and 400 with naïve rabbit serum. A similar dynamic curve of neutralization infection was obtained at both 40 PFU/30,000 cells and 400 PFU/30,000 cells; virus neutralization decreased as serum NAb concentration decreased. When a lower PFU/well ratio was used, the correlation decreased with the presence of samples with high NAb titers to VTT. In this assay, the reference rabbit antiserum neutralized rTV-Fluc at PFU/well ratios of 40 and 400 with NT_50_ of 2336 and 3468, respectively (*P*>0.05). A test serum sample was assayed three or more times with two concentrations of rTV-Fluc to confirm the consistency of the assay. An infectious dose of 400 PFU (MOI = 0.0133), which is the middle of the linear range, was chosen as the optimal viral infection dose to perform the assay due to a lower coefficient of variation (*P*<0.05). This dose was determined to be large enough to produce a high signal and thus permit the evaluation of reduction in transgene expression following neutralization and was also low enough to render the assay sensitive. The final format of the rTV-Fluc neutralizing assay for testing human sera is described in Table 1.

**Table 1:** Description of the final format of the luciferase neutralization assay for human sera.

Step	Description
Viral neutralization, infection of cells and replication	
Samples collection	Collection of blood sample by venipuncture. Inactivation of separated sera at 56°C for 30 min.
Dilution of sera	Preparation of seven 3-fold dilution steps in a flat-bottom 96-well plate in a final volume of 100 ul. Preparation of 150 µl medium for non-neutralized virus control. A control serum with established NT titer should be included in each assay plate as a standard.
Administration of virus and neutralization	Dispensing of 50 µl containing 400 pfu of VTT to each well. Incubation at 37°C, 5% CO_2_ for 1 h.
Addition of cells and viral replication	Addition of 100 µl containing 30,000 Vero cells to each well. Incubation at 37°C, 5% CO_2_ for 24 h.
Luciferase assay	
Addition of substrate	Aspiration of 100 µl supernatant. Addition of 100 µl/well of Bright-Glo^TM^ luciferin equilibrated to room temperature. Incubation at room temperature (20°C) for 2 min, keeping from light.
Measurement of luminescence	Measuring luminescence with the GLOMAXTM 96 microplate luminometer.

### The rTV-Fluc neutralization assay was able to detect NAbs in rabbit, mouse, and human sera

**Figure 2:**
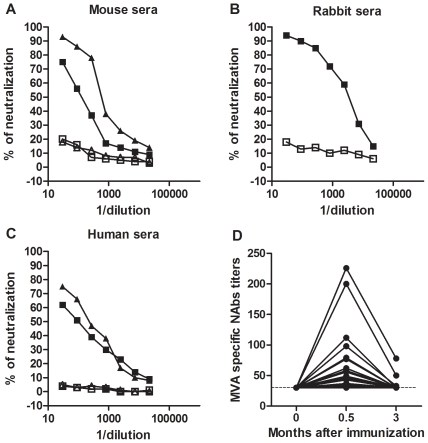
Neutralizing antibodies in mouse, rabbit, and human serum samples. (A) Sera from two rTV-HIVgp 145-vaccinated mice (▪, ▴) and two naïve mice (□,△). (B) An anti-VTT rabbit serum (▪), together with an unrelated rabbit control serum (□). (C) Sera from two rMVA-HIVgpe-vaccinated human (▪, ▴) and two naïve human (□, △) were tested using the rTV-Fluc neutralization assay. The x-axis indicates serial dilutions of sera, ranging from 1∶30 to 1∶21870. (D) The dynamics of anti-rMVA-HIVgpe antibody titers. Two months after the first survey at 15 days after immunization, anti-rMVA-HIVgpe antibody titers of 27 participants were measured again. The x-axis indicates months after immunization. The y-axis indicates the MVA specific neutralization antibody titers. The dotted line represents the detection limit of the assay. Each data point in A-D represents the average of three experiments.

BALB/c mice were vaccinated twice with recombinant rTV-HIVgp 145 vaccine, containing the *env* gene from B’/C recombinant HIV-1 S939, and at three weeks after the second administration, sera were collected and analyzed using the rTV-Fluc neutralization assay (Figure 2A). Neutralizing activity was detected for sera from two vaccinated mice (50% neutralization titer (NT_50_) of 126 and 583), while the two naïve mouse sera showed no significant neutralizing activity. No significant neutralizing activity was observed for the naïve rabbit serum; however, rabbit serum collected at two weeks after the second VTT inoculation was able to neutralize rTV-Fluc with an NT_50_ of 3468 (Figure 2B). Finally, sera obtained from two humans immunized with an experimental HIV vaccinia vaccine (recombinant modified vaccinia Ankara, rMVA-HIVgpe) from the Guangxi Center for Disease Control (CDC) were used to detect neutralizing activity against the heterologous rTV-Fluc virus (NT_50_ of 98 and 226) and no detectable neutralizing activity was shown in two naïve human sera (Figure 2C). An additional 27 rMVA-HIVgpe immunized individuals’ sera were analyzed for neutralizing antibodies. As shown in Figure 2D, the peak levels of specific anti-MVA NAbs were detected as early as two weeks after immunization, then declined rapidly to undetectable levels in most vaccinees after three months. Taken together, these data showed that homologous and heterologous anti-VACV NAbs from various species could be detected using the developed assay.

### Comparison of Fluc and PRNT Vaccinia-specific neutralization assays

**Figure 3:**
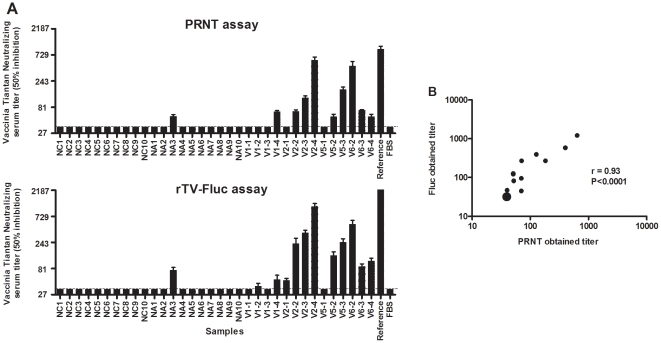
Comparison of two different neutralization assays for the presence of NAbs against VTT in human sera. Serum titers were determined by the dilution at which 50% of plaque viability or luciferase expression was observed. (A) The y-axis indicates the serum dilution at which 50% infection inhibition was observed, relative to the maximum control value. Dotted lines indicate the lower limits of detection as defined by the maximum concentration of serum. NC1 to NA10, human samples; V1-1 to V6-4, mouse samples. (B) Correlation between neutralization titers obtained in luciferase (y-axis) and PRNT (x-axis). Notably, the overstriking point in B represented the 23∶sera with negative titers. Each data point and column represented the average of three experiments.

As part of the characterization of the new Vaccinia neutralization assay, it was important to compare the data obtained with those generated by PRNT. Serum titers obtained by transgene expression inhibition and replication inhibition with plaque scoring were compared. Maximum virus infection was observed in control wells without serum; VTT-antibody positive (reference) serum and FBS alone were included as positive and negative controls, respectively. Comparison of titers revealed that NAb titers of 20∶human and 14∶rTV-HIVgp∶145 immunized mouse sera in the Fluc assay were higher than for the PRNT assay (*P* = 0.12) (Figure 3A). Moreover, two serum samples (V1-2 and V2-1), negative for NAb titer in PRNT, were determined to be positive by the Fluc assay (NT_50_ of 35 and 47). Analysis of the data using the *t*-test (Figure 3B) indicated that under the experimental conditions developed, a high correlation existed between the two methods (r = 0.93, *P*<0.0001). Thus, we established that the newly developed luciferase reporter-gene assay was more rapid (24∶h) and as sensitive as the traditional PRNT.

### Applications of the Fluc neutralization assay in screening of human samples from China

One of the important applications of the developed assay was its use to investigate the seroprevalence of preexisting VTT NAbs in the general population. NAb titer was determined by the Fluc assay in three different age-related cohorts that covered the period from before 1970 to after the cessation of routine smallpox vaccination in China in 1980. As shown in Table 2, all 222 sera from individuals born after 1980 were determined to be negative for anti-VTT NAb titers. The overall percentage of people with positive anti-VTT NAb (titer >30) was 7.6% in individuals born before 1980 (21/278). The percentage of positive VTT NAbs (NT_50_ of >30) and individuals with high titer of anti-VTT antibodies (NT_50_ of >100) increased with age. Of the 31–40 and 41–56 age groups as of 2011, 5.5% and 10.3%, respectively, had evidence of VTT-specific NAbs. There was no significant difference (*P*>0.05) between the two cohorts in the seropositivity rate determined.

**Table 2:** Epidemiological study of neutralizing antibodies in the general population in China.

		VTT NAb titer^a^, num (%)			Total num (%)
		<30 (Neg)	30–100	>100	>30 (Pos)
Age, years	Num				
18–30	222	222 (100%)	0 (0%)	0 (0%)	0 (0%)
31–40	162	153 (94.5%)	8 (4.9%)	1 (0.6%)	9 (5.5%)
41–56	116	104 (89.7%)	10 (8.6%)	2 (1.7%)	12 (10.3%)^b^
Total	500	479 (95.8%)	18 (3.6%)	3 (0.6%)	21 (4.2%)
Sex					
Male	278	266 (95.7%)	10 (3.6%)	2 (0.7%)	12 (4.3%)
Female	222	213 (96.0%)	8 (3.6%)	1 (0.4%)	9 (4.0%)
Total	500	479 (95.8%)	18 (3.6%)	3 (0.6%)	21 (4.2%)
Site					
Beijing	300	290 (96.6%)	8 (2.7%)	2 (0.7%)	10 (3.3%)
Anhui	200	189 (94.5%)	10 (5.0%)	1 (0.5%)	11 (5.5%)
Total	500	479 (95.8%)	18 (3.6%)	3 (0.6%)	21 (4.2%)

a The titers (vaccinia-neutralizing activity) were determined using the neutralization assay described in Table 1. Arbitrary intervals were established as a qualitative reference for the potency of a determined serum, i.e., negative (<30), low (30–100), and high (>100).

b the percentage of positive VTT NAbs increased with age.

Taken together, these data showed that the developed assay was sensitive enough to detect even low levels of viral inhibition (NT_50_ of 30–100), and most neutralization titers detected in 21 seropositive samples were in this range (Table 2). Interestingly, it was found that even >40 years post-vaccination, one individual maintained a high level of Vaccinia-specific NAbs, with a titer similar to that obtained with the anti-VTT rabbit sera (ATRS) (NT_50_ of 1810 vs 3468). There was no statistically significant association observed between gender and the presence of antibodies against VTT (*P*>0.05). We further analyzed the samples collected from various locations, including Beijing and Anhui provinces, and found no significant difference in the seropositivity rate of NAbs.

## Discussion

In this study, we established a novel VACV-neutralization assay to replace the PRNT, which was based on inhibiting the infection of Vero cells by a recombinant VTT that carried a firefly luciferase gene with the resulting inhibition of the transgene expression. The quantitative analysis using the assay was supported by data that showed the number of rTV-Fluc particles bound to cells was directly proportional to transgene expression [22]. The use of Fluc as a reporter offers several advantages compared to the standard PRNT as follows: (i) higher resolution available due to the precise measurement of firefly luciferase transgene expression and the amplification effect of chemiluminescence; (ii) greater reliability since the data gathering step of the rTV-Fluc assay is automated; (iii) shorter overall assay time due to a reduction in incubation time (24 h vs. 4–6 d) [15], [16]; (iv) higher throughput via the 96-well format versus 6-well in PRNT; (v) five-fold reduction in the volume of valuable sera needed for analysis. Recombinant VACV expressing other reporter genes, such as GFP and β-gal, may also be useful for establishing high-throughput, sensitive neutralization assays. Sprangers et al. (2003) compared luciferase activity to the automated quantitation of GFP and β-gal protein expression in determining anti-Ad5 NAb, and found that luciferase activity detection was more sensitive than other transgene systems and required fewer target cells, which made it suitable for use in 384-well plates for high-throughput [20]. Hatakeyama et al. (2005) noted only moderately positive correlation between antibodies detected by the PRNT assay and those by ELISA among representatives of Japanese population, which was due to a number of epitopes on the viral proteins other than neutralizing epitopes on Vaccinia virus [23].

The current study examined the importance of the standardization of critical parameters including the choice of cell line, viral infection dose, incubation time, and washing steps that could affect the sensitivity, reproducibility, specificity, and correlation with the PRNT assay. Adequate control of these parameters reduces variability in titer values and allows reliable comparison of data from different studies [22]. Cells were chosen based on their ability to allow rTV-Fluc replication and to not produce cytopathic effect (CPE) following a 24–48 h incubation. Two cell lines (Vero and 143TK) and primary chicken embryo fibroblasts (CEF) were tested for compatibility with the rTV-Fluc assay. Of the three, Vero cells allowed viral replication after 24 h without visible CPE, and the results obtained with Vero cells were reproducible. The determination of optimal viral concentration is important because the infectious dose must be low in order to increase assay sensitivity. However, the infecting dose should also be high enough to produce a signal that will allow measurement of reduction due to neutralization.

In this assay, the dynamic range was found to be 22–1800 PFU/30,000 cells. We determined that 400 PFU (MOI = 0.0133), which was in the middle of the linear range, was appropriate for both reproducibility and sensitivity. The linear correlation obtained between the Vaccinia vector concentration and transgene Fluc expression supports the hypothesis of one virion-one transduction event [24]. It was found that sera from three different species had different levels of nonspecific inhibition effect at 1∶30 dilution. In this assay, the samples were considered positive only when the inhibition percentage reached 50%. In fact, all naïve samples at the starting dilution (1∶30) were determined to be negative although their inhibition percentage ranged from 20–40%. Thus, an inhibition percentage <50% will not affect the results of detection. It was found that the nonspecific inhibition effect decreased as serum dilution increased from 30 folds. So, we determined that the nonspecific neutralization effect could be ignored when the serum dilution was more than 30 folds.

In order to test the rTV-Fluc assay, 20 sera from healthy human individuals born before 1980, and 14 rTV-HIVgp 145 immunized mouse sera at two weeks after second immunization were tested. Comparison of the neutralization titers obtained by rTV-Fluc to those by PRNT indicated a high correlation (r = 0.93). Moreover, statistical comparisons indicated that the limit of detection of rTV-Fluc assay was lower than that of the PRNT assay. We believed that the slightly enhanced sensitivity observed was due to differences in the protocols followed. For both assays, the virus was incubated with antibody for 1 h, however, in the rTV-Fluc assay the virus-antibody suspension was only diluted two-fold when the cells were added and was not removed from the incubation. In contrast, antibodies were removed after 2 h pre-incubation in the PRNT, when the liquid medium was replaced with a semi-solid overlay in order to obtain discrete plaques. Neutralization of VACV could occur even after the virus had attached to cells [25], and hence, the new method described here was sensitive enough to detect both high-titer and low-titer NAbs in mouse, rabbit, and human serum samples, and should provide simple validation and transfer to other laboratories.

The present study investigated the utility of the rTV-Fluc neutralization assays in two different studies. In the first study, the assay was used to perform an epidemiological study to ensure that VTT was the optimal vaccine vector for application in the Chinese populations. There were concerns that the utility of recombinant VACV vector-based vaccines could be limited by the high prevalence of pre-existing VACV NAbs in human populations [13]. Now, live-virus vector-based vaccination strategies are likely to increasingly use VACV-derived vectors in future clinical trials [26]–[33]. Indeed, VTT was successfully used as virus vector for the development of HIV, HBV, HPV, H5N1 AIV, SARS-CoV, and Rabies virus vaccines, conferring protection to immunized animals [5]–[11]. However, significant problems remain for the use of VTT vectors in humans, such as preexisting immunity to viral vectors. The pre-existing immunity of the vaccinee against the viral vector is likely to limit the amount of virus that can reach and infect the target tissue, thus, decreasing the expression of the virus encoded foreign antigen, and consequently the immune response generated against the target antigen [13], [34].

It is known that the Orthopoxvirus serological tests, such as ELISA, PRNT, are not virus species specific due to the high level of cross-antigenicity observed among the species of the OPXV genus. In this study, the specificity of rTV-Fluc assay was not analyzed with other orthopoxvirus. However, we determined that the antibodies detected with developed assay most likely had been induced during smallpox eradication campaign by VTT, since no other strain had been used for vaccination against smallpox in China. To our knowledge, this was the first study of the prevalence and potency of VTT-specific NAb in human samples from different geographic origin in China. The serologic survey was performed using a diverse population (60% in Beijing, others in Anhui) with a male-to-female ratio of 1∶1, comprising a wide range of ages (18–56 years of age as of 2011). Only a low prevalence of NAb to VTT (7.6%) was seen in Chinese people born before 1980, mostly at low titers, and these results did not corroborate immunological studies and VACV specific antibodies population screenings that indicated anti-VACV NAb were low and stable in vaccinees for up to 30–70 years post-vaccination [12], [35], [36]. In Japan, Shuji Hatakeyama et al. [23] reported that approximately 80% of people born before 1969 and 50% of those born between 1969 and 1975 were also found to have maintained NAb against VACV.

Interestingly, we also found that the percentage of positive VTT NAbs (titers >30) in China increased with age. We believed that this may be due to the different number of smallpox vaccinations participants had received. The prevalence of pre-existing NAb to VTT could guide the future administration of VTT vector-based products in Beijing and Anhui provinces.

Since the kinetics of immunity to foreign antigen seemed to parallel the kinetics to vector [37], the second part of the study focused on the evaluation of the dynamics of anti-VACV NAb titers generated upon the administration of recombinant MVA vector-based vaccines in humans. The rTV-Fluc neutralization assay described here was able to detect the specific humoral responses elicited by the MVA vectors in a time- and dose-dependent manner. Although the duration of NAbs with multiple MVA vector vaccination was not analyzed in present study, it was believed that the stronger and long-lasting immunity could be achieved by the prime-boost strategy in the current Chinese population, as indicated by Lai L et al. [38].

Amanna et al. [39] reviewed that the smallpox vaccine-induced antibody responses were both necessary and sufficient for protection against lethal monkeypox infection. At present, the duration of Vaccinia-specific NAbs is controversial. Some studies have demonstrated that full protective immunity conferred by smallpox vaccination lasted only 3–5 years and that even partial immunity faded substantially after 10–20 years [40]–[42]. Meanwhile, others have shown that immunity to smallpox may still be present many years after vaccination [23], [35], [43]. Several studies have shown that serum neutralizing antibody titers >32 were associated with protective immunity against smallpox disease [16], [42], [44], [45]. Using this level of NAb as a marker for smallpox resistance, we determined that only 7.6% of the subjects born before 1980 in China demonstrated titers above this level. We do not believe that the low prevalence of VTT NAb was due to low sensitivity of our assay. The sensitivity of rTV-Fluc assay was similar and even slightly higher to the PRNT. Until now, there has been no other report on the epidemiological study of VACV-specific NAbs in the general population of China. Therefore, it was unknown whether this limited study population was truly representative of national level of antibody titers. This will be the focus of future studies. These data suggested that people born after the early 1980s, when China stopped vaccinating against smallpox, would be vulnerable to smallpox, and the vaccinated older population would also be vulnerable due to low NAb titers. Some previous findings had indicated that human memory B cells could be maintained for life in the absence of antigenic re-exposure [39]. Although the VACV NAbs in this study declined to undetectable levels in most populations studied in China, we believed that VACV-specific memory B cells could be activated to rapidly produce neutralizing antibodies upon reimmunization. Therefore, VACV and the recombinant VACV vector-based vaccines should attract more attention for their use as a vaccine against Variola virus in China, due to the rise in bio-terrorism threats around the world [46]. In the event of an Orthopoxvirus outbreak, the speed and high-throughput nature of the rTV-Fluc assay may prove extremely valuable.

In summary, a simple, objective, reproducible, time- and labor-saving chemiluminescence-based neutralization assay was developed for the determination of anti-VACV NAbs titers for both human and animal sera. This new assay may be a good candidate method for not only performing epidemiologic surveys but also for monitoring the anti-VACV immune responses in large-scale clinical trials. Moreover, the assay could also be used in basic research since it gives a direct measurement of neutralizing activity.

## Methods

### Ethics issues

The ethics issues in the study was inspected and approved by Ethical Committee of Beijing Blood Center. Each participant was informed of the purpose of the study and written consent was also obtained from each participant involved in this study.

### Cells and virus

Vero (ATCC, CCL81) and 143TK (CCTCC, GDC076) cells were used for the Fluc neutralizing assay, grown at 37°C under 5% CO_2_ in Dulbecco’s modified Eagle’s medium (DMEM) supplemented with 10% fetal bovine serum (FBS), 1% L-glutamine, 0.5% combined antibiotics and 1% non-essential amino acids (HyClone, South Logan, UT, USA). Primary chicken embryo fibroblasts (CEF) prepared from 9-day-old embryos were grown in DMEM supplemented with 10% FBS. The original VTT strain was derived in our institute. VTT was propagated in CEF cell, and the titer was determined by a plaque-forming assay using crystal violet staining and primary CEF [1].

### Vaccinia virus antisera

For experiments using the VTT, sera from mice and rabbits vaccinated with VTT and control normal mouse and rabbit sera were used. The anti-VTT rabbit sera (ATRS) was kindly provided by Dr. Huanyu An (Beijing Tiantan Biological Products, Beijing, China). Samples from recombinant MVA-HIVgpe virus-vaccinated participants were kindly provided by Dr. Wei Kong (Jilin University, Changchun, China).

### Normal human serum samples

Serum samples from 278 donors (169 males, 109 females) ranging in age from 31–56 years as of 2011 were obtained from a diverse human population of healthy individuals in Beijing and Anhui province, China, with a known history of smallpox vaccination. Negative controls included 222 serum samples that had been obtained from unvaccinated people (109 male, 113 female) born after 1980 (18–31 years, as of 2011) to evaluate the specific NAbs against VTT since routine smallpox vaccination was discontinued in China in 1980. The samples from healthy participants were collected from the Beijing (300 samples) and Anhui province (200 samples). All samples were frozen at –80°C and banked at the various institutions after collection. Samples were shipped in dry ice to our department in the National Institutes for Food and Drug Control. All samples were thawed, aliquoted, and stored at –80°C. All samples in our study were subjected to identical treatment and storage conditions and were considered of equivalent quality.

### Construction of rTV-Fluc

PCR was used to construct recombinant shuttle plasmid pSCFluc [28], [47]. Briefly, the firefly luciferase gene of *Photinus pyralis* was from pLUCF (kindly provided by John T. Schiller, National Cancer Institute, Bethesda, MD, USA) and inserted into the SalI-SmaI site of pSC65 transfer vector (kindly provided by B. Moss, NIAID, NIH, USA), under the control of the VACV-specific early/late promoter, and adjacent to the gene that encodes *lacZ* expressed as a screening marker for recombination, which was regulated by the VACV P7.5 late promoter. Primers were designed with the inclusion of restriction enzyme sites for SalI or SmaI. The two primers used for this test were: 5’-GTCGACGCCACCATGGAAGATGCCAAAAAC-3’ (sense, SalI site underlined) and 5’-CCCGGGTTACACGGCGATCTTGCCGCCC-3’ (antisense, SmaI site underlined). The amplification cycles were 94°C for 2 min followed by 35 cycles of 94°C for 30 s, 55°C for 30 s and 72°C for 2 min plus the last extension of 72°C for 10 min. Amplified PCR products were purified using a QIAquick PCR purification kit (QIAGEN, Valencia, CA, USA) and were subjected to direct DNA sequencing using an automated ABI 377 DNA sequencer (Applied Biosystems, Foster City, CA, USA). The rTV-Fluc virus was generated in CEF cells using a homologous recombination method [48]. CEF cells were infected with VTT at multiplicity of infection (MOI) of 0.01 and subsequently transfected with the shuttle vector, pSCFluc, designed to recombine specifically with the TK gene of the VTT to obtain rTV-Fluc recombinant viruses, which were selected by blue/white plaque screening. The rTV-Fluc virus was propagated, purified, and titrated in primary CEF, as previously described [48].

### Firefly luciferase-based vaccinia neutralization assay (Fluc assay)

Neutralization was measured by the reduction in Fluc reporter gene expression of rTV-Fluc inhibited by Vaccinia NAb present in serum samples. During the development stages of the assay, the following parameters were tested: cell lines, incubation time, cell density, washing with PBS, and virus concentration. Different cell lines (Vero and 142TK) and primary CEF were screened to determine the infection profiles of the rTV-Fluc virus. All cells were added as a trypsinized single-cell suspension. Viral progeny were allowed to grow in the cells for up to 48 h after infection. Cells density was tested with 10^4^ and 3×10^4^ cells/well in a 96-well flat-bottom culture plate (Corning-Costar, Tokyo, Japan). The influence of washing once with PBS on luciferase quantitation was tested with serial titers of rTV-Fluc virus. In determining the optimal viral dose for the assay, we considered both the natural infectivity of the virus in Vero cells and the variability of infection in the presence of varying dilutions of serum containing vaccinia NAb involving 0.8, 2, 7, 22, 66, 200, 600, 1800, and 5400 PFU/50 µL of diluted virus and nine dilutions of anti-VTT rabbit serum. Diluted serum (100 µL) was distributed into each of three corresponding wells in a 96-well plate and 50 µL of rTV-Fluc virus was added to each well and the plates were incubated at 37°C, 5% CO_2_ for 1 h. Following incubation, 100 µL of cells was added to each well. Plates were placed on a plate shaker for 1 min and incubated at 37°C, 5% CO_2_ for up to 48 h. After incubation, 100 µL of supernatant was aspirated and 100 µL of D-luciferin substrate (Caliper, Hopkinton, MA, USA) was added into each well protected from light at room temperature for 2 min, after which luminescence was measured using a GLOMAX 96 microplate luminometer (Promega, Madison, WI, USA). An anti-VTT rabbit serum (ATRS) was used as reference to establish an in-house standard serum sample, whereas pooled serum samples from Vaccinia-naïve individuals, pre-immune animal serum samples, and FBS were used as negative controls. The 50% neutralization titer (NT_50_) for each serum sample was defined as the serum dilution at which the relative light unit (RLU) was reduced by 50% compared with virus-containing control wells after subtraction of the background RLU in cell-containing control wells. Titers of >30 were considered positive.

### Plaque reduction neutralization test (PRNT)

The traditional control neutralization assay was performed according to an optimized protocol similar to that previously described [23]. Sera were serially diluted to five five-fold dilutions at the range from 1∶40 to 1∶25000, depending on the expected titer. Tubes containing 550 µL of each dilution and an equal volume of rTV-Fluc virus (400 PFU/mL) were mixed. After incubation at 37°C, 5% CO_2_ for 1 h, 500 µL was added to Vero cells, in duplicate, and plates were incubated for 2 h at 37°C, 5% CO_2_ with intermittent rocking, followed by the addition of 3 mL of overlay medium containing 0.5% agarose. On day 4, viral plaques were visualized by staining with 0.1% crystal violet in PBS containing 0.2% formaldehyde and counted. The NT_50_ was defined as the reciprocal of the serum dilution required for 50% reduction in rTV-Fluc plaques.

### Statistical analysis

The neutralization titers of sera tested by both PRNT and luciferase were compared. The variance between average titers obtain by both methods and the correlation coefficient were calculated using Student’s *t*-test. Comparisons between the VTT seroprevalence in different groups were evaluated using Chi-square test, and statistical analyses were computed with GraphPad Prism 5.0 (GraphPad Software, San Diego, CA). For all analyses, *P*<0.05 was considered significant.
